# Protocol for *in vivo* chromatin immunoprecipitation on purified chromatin isolated from mouse liver nuclei

**DOI:** 10.1016/j.xpro.2025.103616

**Published:** 2025-01-31

**Authors:** Lei Li, May G. Akl, Scott B. Widenmaier

**Affiliations:** 1 Department of Anatomy, Physiology, and Pharmacology, University of Saskatchewan, Saskatoon, SK, Canada

**Keywords:** molecular biology, antibody, chromatin immunoprecipitation, ChIP

## Abstract

Chromatin immunoprecipitation (ChIP) is used to investigate genome binding by transcription factors, but it can be problematic. We present a protocol to isolate fixed DNA-protein complexes from mouse liver prior to chromatin shearing. We describe steps for liver disaggregation and cross-linking, DNA-protein complex isolation, chromatin shearing, and quality control analysis as well as procedures for ChIP, DNA purification, and ChIP analysis. This protocol yields high-quality samples using commercial antibodies.

For complete details on the use and execution of this protocol, please refer to Akl et al.^1^

## Highlights

• Steps for conducting ChIP on *in vivo* mouse liver samples
• Protocol for purifying fixed DNA-protein complexes from enriched nuclear fractions
• Instructions for chromatin shearing and performing quality control analysis
• Detailed procedure for analyzing samples after high-quality ChIP

## Abstract (editor highlights)

Publisher’s note: Undertaking any experimental protocol requires adherence to local institutional guidelines for laboratory safety and ethics.

## Graphical abstract

**Figure:**
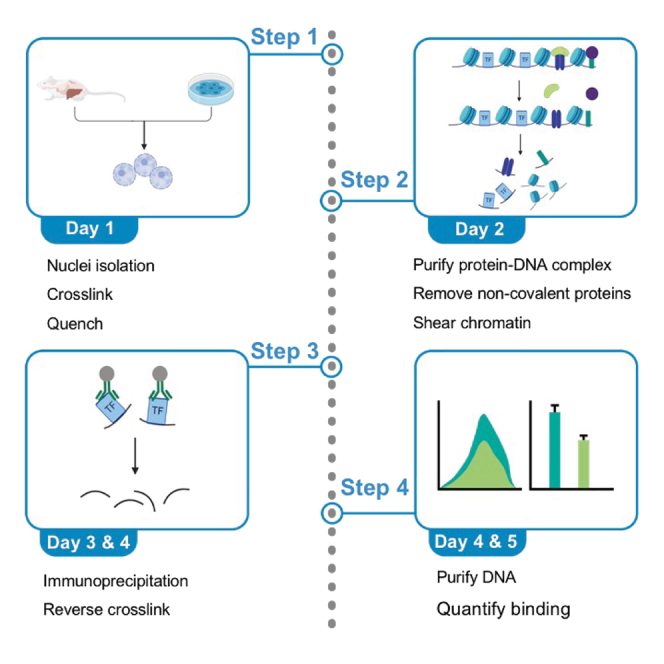

## Before you begin

The protocol described here has been optimized for mouse liver tissue. Here, we describe specific steps modified from Qin & Wang^2^ using mouse liver tissue, for which we have found can be utilized in ChIP-sequencing (ChIP-seq) and -quantitative polymerase chain reaction (ChIP-qPCR) experiments to identify the genomic sites that are bound by two transcription factors named nuclear factor erythroid 2 related factor-1 (NRF1) and nuclear factor erythroid 2 related factor-2 (NRF2).^1^ In our experience, this protocol renders high yield and quality using anti-NRF1 and anti-NRF2 antibodies. Though not described here, this protocol is also effective using nuclei isolated, beginning at step 2e, from the following cells grown in culture: Hep3b human hepatoma cells, Hepa 1–6 mouse hepatoma cells, and primary mouse embryo fibroblast (MEF) cells. While our focus here is on *in vivo* mouse liver samples, we mention important differences when using cell culture samples.

**Figure 1:**
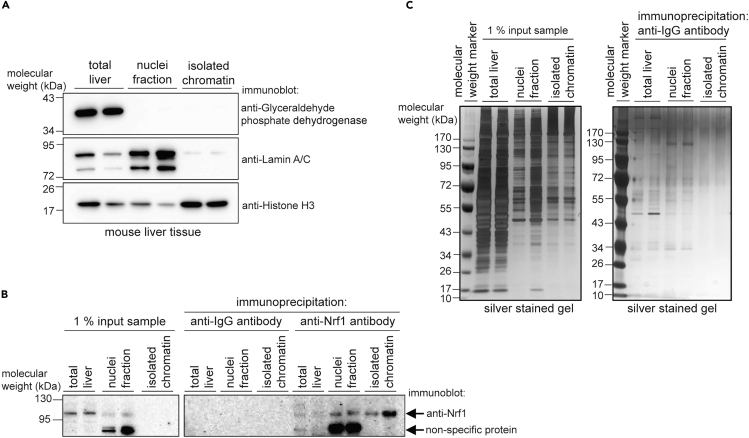
Fraction analysis (A–C) The enrichment of nuclei and chromatin subcellular fractions from total liver tissue isolated from mice was assessed by immunoblot of these fractions (A) and by immunoblot on anti-IgG control and anti-Nrf1 immunoprecipitated samples (B) as well as by performing a silver stain of the NuPAGE gels used for electrophoresis (C). For these representative liver samples, total liver lysate and nuclei fraction were prepared using the method we described previously.^1^ Chromatin was prepared by using DNAzol, as described in this paper.

We use about 500 mg liver tissue for ChIP experiments for transcription factors of interest (NRF1 and NRF2). DNA yield is approximately 250–420 μg when using DNAzol^3^ reagent to isolate chromatin, which has more enriched chromatin proteins (Figure 1A), higher yield immunoprecipitation products (Figure 1B) and less background (Figure 1C) compared to nuclear fraction. For cultured cells, we normally get 200–300 μg of DNA from 1–2 × 10^7^ cells. We typically use 30 μg of DNA for each ChIP reaction. However, less DNA can be used for each ChIP to achieve sufficient enrichment if working with more effective targets, such as histone proteins or highly abundant transcription factors with high quality antibodies.

### Institutional permission

This study was approved by the University of Saskatchewan’s Animal Care Committee, in accordance with animal use protocol number AUP20180090.

#### Preparing buffers

**Timing: 2–3 h**

1. Prepare the buffers listed below (recipes are listed below in materials and equipment section), making sure to adjust the temperature as indicated.
  a. 250-STM (sucrose/tris/MgCl2) buffer (homemade, filtered, 4°C).
  b. 16% formaldehyde (W/V), methanol-free (Cat# 28908, Thermo Fisher Scientific. Ready-to-use, room temperature).
  c. 2.5 M glycine (homemade, filtered, room temperature).
  d. Phosphate buffered salts (PBS) buffer (Cat# SH3025601, Ge Hyclone, 4°C).
  e. 100% ethanol (Cat# 106-02-17N, Commercial Alcohols, 4°C).
  f. 75% ethanol (homemade, 4°C).
  g. Immunoprecipitation (IP) buffer (homemade, filtered, 4°C).
  h. 10% sodium dodecyl sulfate (SDS), (homemade, filtered, room temperature).
  i. 5 M sodium chloride (NaCl) (homemade, room temperature).
  j. 3 M sodium acetate, pH 5.2 (homemade, filtered, 4°C).
  k. 2× ChIP elution buffer (filtered, freshly made, room temperature).
  l. 100 mM phenylmethylsulfonylfluoride (PMSF) in isopropanol (homemade, −20°C).
  m. 100 mM dithiothreitol DTT (homemade, −20°C).
  n. Protease inhibitor cocktail solution (Cat# 3115879001, Roche 50×, −20°C).

**CRITICAL:** All marked solutions should be filtered by a 0.22–0.45 μm filter unit.

## Key resources table

**Table:** 

REAGENT or RESOURCE	SOURCE	IDENTIFIER
**Antibodies**		

Anti-NRF1 (2 μg/mL for ChIP and 1:1,000 for immunoblot)	Cell Signaling Technology	Cat# 8052; RRID: AB_11178947
Anti-NRF2 (2 μg/mL for ChIP and 1:1,000 for immunoblot)	Cell Signaling Technology	Cat# 12721; RRID: AB_10891429
Anti-GAPDH (1:1,000)	Invitrogen	Cat# AM4300; RRID: AB_2536381
Anti-Lamin A/C (1:1,000)	Cell Signaling Technology	Cat# 4777; RRID: AB_10545756
Anti-rabbit HRP conjugate (1:5,000)	Cell Signaling Technology	Cat# 7074S; RRID: AB_2099233
Rabbit (DA1E) mAb IgG (2 μg/mL)	Cell Signaling Technology	Cat# 3900; RRID: AB_1550038

**Chemicals, peptides, and recombinant proteins**		

16% Formaldehyde (W/V), methanol-free	Thermo Fisher Scientific	Cat# 28908
ChIP-Grade Protein G Magnetic Beads	Cell Signaling Technology	Cat# 9006
DNAzol	Invitrogen	Cat# 10974020
Nitrocellulose membrane	Bio-Rad	Cat# 162-0115
NP-40 Alternative	Calbiochem	Cat# 492016100ML
NuPAGE 4%–12% Bis-Tris Protein Gels	Thermo Fisher Scientific	Cat# NP0336
NuPAGE MOPS running buffer	Thermo Fisher Scientific	Cat# NP0001
Phosphate-buffered saline (PBS)	GE HyClone	Cat# SH3025601
Protease inhibitor cocktail	Roche	Cat# 11873580001
Proteinase K	Roche	Cat# 3115879001

**Critical commercial assays**		

GeneJET PCR Purification Kit	Thermo Fisher Scientific	Cat# K0702
PowerUp SYBR Green Master Mix	Thermo Fisher Scientific	Cat# A25742
Super Signal West Femto maximum sensitivity substrate	Thermo Fisher Scientific	Cat# 34096

**Deposited data**		

ChIP-seq data from Akl et al. (2023)^1^		NCBI; BioProject ID: PRJN806849

**Experimental models: Organisms/strains**		

Adult male and female *Mus musculus* C57BL/6J at 9–12 weeks of age	The Jackson Laboratory	Jax: 000664

**Oligonucleotides**		

*Ces1g* Forward: CATTGCAGCATCAGCCTGT Reverse: TGGCCTGAGACAAAGTTCTGAG	N/A	Akl et al. (2023)^1^
*Gstm1* Forward: GGGAACAACAAGCGAATCGG Reverse: GTATTCAGCCAGGGTGCAGT	N/A	Akl et al. (2023)^1^
*Psma1* Forward: GGGCTCAACACACCATAGCTC Reverse: ACGATCACCGAACCGTAGTT	N/A	Akl et al. (2023)^1^
*Psmc2* Forward: CCTTCCACCAGACAACCCTT Reverse: CGCTGGTGTCTTCCCTTTCT	N/A	Akl et al. (2023)^1^

**Software and algorithms**		

Fiji open-source image analysis software	Fiji	RRID: SCR_002285; https://fiji.sc/
GraphPad Prism 9.3.1	GraphPad Software	RRID: SCR_002798; https://www.graphpad.com

**Other**		

Bioruptor Pico tube, 1.5 mL	Diagenode	Cat# C30010016
Bioruptor pico sonicator device	Diagenode	Cat# B01060010
(Alternative) CPX130 Ultrasonic Processor	Cole-Parmer instruments	N/A
CFX384 Touch Real-Time PCR Detection System	Bio-Rad	N/A
Dissecting scissor	Almedic Ltd	36-104-0098
DynaMag-2 magnetic stand	Thermo Fisher Scientific	Cat# 12321D
Centrifuge tube with cap, 1.5 mL	Axygen	Cat# 14-222-672
MicroAmp Clear adhesive film	Thermo Fisher Scientific	Cat# 4306311
Microplates, PCR, 384-well skirted	Axygen	Cat# 14-222-306
NanoDrop spectrophotometer	Thermo Fisher Scientific	Cat# ND-ONE-W
Teflon pestle tissue grinder	Wheaton Kimble	Cat# 358009
Power homogenizer	Wheaton	Cat# 903475

## Materials and equipment

**Table:** 250-STM buffer

Reagent	Final concentration	Amount
Sucrose	250 mM	42.78 g
1 M Tris-HCl pH 7.4	50 mM	25 mL
1 M MgCl_2_	5 mM	2.5 mL
ddH_2_O		To 500 mL
**Total**	N/A	**500 mL**

Filter and store at 4°C for up to 1 month.

**Table:** IP buffer

Reagent	Final concentration	Amount
NaCl	150 mM	4.383 g
1 M Tris-HCl pH 7.4	50 mM	25 mL
100 mM EDTA	5 mM	25 mL
10% NP-40	0.5% vol/vol	25 mL
10% Triton X-100	1.0% vol/vol	50 mL
ddH_2_O		To 500 mL
**Total**	N/A	**500 mL**

Filter and store at 4°C for up to 3 months.

**Table:** Protein denature buffer

Reagent	Final concentration	Amount
1 M Tris-HCl pH 7.4	50 mM	2.5 mL
10% SDS	2%	10 mL
Urea	8 M	24.024 g
ddH_2_O		To 50 mL
**Total**	N/A	**50 mL**

Freshly made.

***Note:*** To dissolve faster, this buffer can be incubated at 37°C water bath with gentle mixing occasionally.

**Table:** 2× ChIP elution buffer

Reagent	Final concentration	Amount
NaHCO_3_	200 mM	84 mg
10% SDS	2%	1 mL
ddH_2_O		4 mL
**Total**	N/A	**5 mL**

Made freshly and stored at room temperature.

• 100 mM PMSF/isopropanol:
  Add 174 mg PMSF in 10 mL isopropanol.
  Aliquot and store at −20°C for up to 6 months.
• 10% SDS:
  Add 10 g SDS in 80 mL MilliQ water, add a magnetic flea and place on a magnetic stirring plate to mix solution. Heat solution to 60°C until SDS powder fully dissolved and top up the solution to 100 mL using MilliQ water.
  Filter and store at room temperature for up to 3 months.

**CRITICAL:** SDS is a fine powder and toxic chemical; it should be weighed under a fume hood to avoid inhalation.

• 2.5 M Glycine:
  Add 93.8 g glycine in 400 mL MilliQ water. Adjust the volume to 500 mL with MilliQ water after glycine completely dissolved.
  Filtered and store at room temperature for up to 3 months.
• 5 M NaCl:
  Add 292 g of NaCl in 800 mL of MilliQ water. Adjust the volume to 1 L with MilliQ water.
  Filter and store at room temperature up to 6 months.
• 3 M sodium acetate, pH 5.2:
  Add 24.6 g of sodium acetate in 70 mL MilliQ water, Adjust the pH to 5.2 by adding glacial acetic acid. Adjust the volume to 100 mL with MilliQ water.
  Filter and store at room temperature up to 6 months.
• Protease inhibitor cocktail solution (50×):
  Dissolve one tablet in 1 mL MilliQ water.
  Aliquot and store at −20°C for up to 3 months.

## Step-by-step method details

### Liver tissue disaggregation and cross-linking

#### Liver tissue was homogenized and nuclei isolated and cross-linked to trap protein-DNA interactions

**Timing: Day 1, 2–3 h**

Fresh mouse liver tissue extraction, homogenization, nuclei isolation, and cross-linking.

1. Prepare solution for homogenizing and cross-linking.
  a. Prepare homogenizing solution by freshly adding 1 mL 100 mM PMSF to 99 mL 250-STM buffer at final concentration of 1 mM. Keep solution on ice. 10 mL will be used for each liver sample.
  b. Prepare cross-linking solution in fume hood by adding 16% formaldehyde to 250-STM buffer at a final concentration of 1% (e.g., add 2 mL 16% formaldehyde to 30 mL buffer). Bring the cross-linking solution to room temperature before use. 10 mL will be used for each liver sample.
2. Mouse liver cross-linking (troubleshooting 1).
  a. Start with 250–500 mg fresh mouse liver tissue, place the liver piece in a 50 mL beaker containing 10 mL ice cold homogenizing solution.
  b. Cut tissue into about 3 mm small pieces with a dissecting scissor, and transfer solution to a Teflon pestle tissue grinder mounted on Wheaton power homogenizer.
  c. Set the speed of homogenizer at 4, disaggregate liver tissue for 5 s to get a homogeneous suspension.
  d. Transfer the tissue suspension into a 50 mL tube and centrifuge at 800 g for 15 min at 4°C. Gently discard the supernatant and keep nuclei-containing pellet.
  e. Resuspend the pellet in 10 mL of 250-STM buffer containing 1% of formaldehyde (cross-linking solution).
    ***Note:*** All steps with formaldehyde need to be done in a chemical hood.
  f. Incubate by gentle mixing on a rotator for 30 min at room temperature.
  g. Stop the cross-linking by adding 500 μL of 2.5 M glycine stock to a final concentration of 125 mM glycine. Continue to rotate at room temperature for 5 min.
  h. Centrifuge samples at 800 g for 15 min at 4°C. Gently discard the supernatant and keep the pellet.
  i. Resuspend the pellet in 10 mL of ice-cold PBS containing 100 μL 100 mM PMSF (Final concentration is 1 mM) to wash the pellet.
  j. Centrifuge at 2000 g at 4°C for 5 min and discard the supernatant.
  k. Repeat step i-j one more time.
    **Pause Point:** Cross-linked liver nuclei samples can be used immediately or stored at −80°C for at least 6 months.
    **CRITICAL:** Formaldehyde will be oxidized to formic acid when exposed to oxygen, which will compromise the cross-linking efficiency. We recommend purchasing small bottles of formaldehyde and preferably finish them within one month. The 16 % formaldehyde used in this protocol is a 10 mL solution in an ampule-sealed package, which allows access to ‘fresh’ formaldehyde each time.
    **CRITICAL:** The cross-linking time is crucial for obtaining optimal result, especially for transcription factors and cofactors. Therefore, additional optimization might be required depending on your protein of interest.

### DNA-protein complex isolation

**Timing: Day 2, 5 h**

Isolating DNA-protein complex by using DNAzol,^3^ which is a guanidine salt containing reagent used for genomic DNA isolation. Qin & Wang^2^ have used this reagent to isolate and investigate crosslinked nuclear acid-binding proteins. The following steps are used to isolate and purify crosslinked DNA-protein complex.

Cross-linked liver nuclei samples are lysed by DNAzol reagent, non-crosslinked protein is dissociated, and DNA-protein complexes are isolated and sheared.

3. DNA-Protein complex isolation.
  a. Add 5 mL DNAzol reagent to the nuclei pellet (or 1 mL per 100 mg homogenized tissue), resuspend the pellet with a wide bore tip.
    ***Note:*** Prepare wide bore pipette tips by cutting 2–3 mm from the end of tips.
  b. Incubate for 15 min at room temperature with gentle rotation.
    **CRITICAL:** Do not clear the sample by centrifugation after this step.
  c. Add 2.5 mL ice-cold 100% ethanol (or half volume of DNAzol Reagent used) to the tube.
  d. Mix samples by inversion and incubate at −20°C for 1 h to precipitate the DNA-protein complexes.
  e. Prepare protein denature buffer during precipitation.
  f. Pellet precipitates via centrifugation at 4000 g at 4°C for 10 min.
  g. Wash pellets with 5 mL ice-cold 75% ethanol (or same volume as DNAzol reagent used).
  h. Centrifuge at 4000 g at 4°C for 10 min.
  i. Discard supernatant and air-dry pellets for 5–15 s.
  j. Add 5 mL of protein denature buffer and resuspend pellets (or same volume as DNAzol Reagent used) and then incubate samples at 37°C for 30 min with gentle shaking to remove non-covalently bound proteins from chromatin sample.
  k. Add 5 mL of 5 M NaCl (equal volume as protein denature buffer) and incubate at 37°C for 30 min.
  l. Add 1 mL (0.1 volume) of 3 M sodium acetate and 30 mL (3 volumes) of ice-cold 100% ethanol.
  m. Incubate at −20°C for 1 h to precipitate purified DNA and DNA-protein complexes.
  n. Collect pellet by centrifugation at 4000 g at 4°C for 10 min.
  o. Resuspend and wash the pellets with 25 mL ice-cold 75% ethanol to remove salts and detergents.
  p. Centrifuge at 4000 g at 4°C for 10 min. Discard supernatant and place samples on ice.
  q. Repeat step o-p two more times to have total of 3 washes.
  r. Air-dry the pellets for 10 min at room temperature.
  s. Dissolve pellets on ice with 5 mL IP buffer containing 0.5% SDS and protease inhibitor cocktail.
    ***Note:*** To make complete IP buffer for dissolving pellets, add 250 μL 10 % SDS, 100 μL 50× protease inhibitor cocktail solution to 4750 μL IP buffer.
    ***Optional:*** Save 50 μL aliquot of each sample as pre-sonication control. Another aliquot can also be saved for verifying target of interest by western blot.

### Chromatin shearing

**Timing: day 2, 2–3 h**

Isolated chromatin is sheared and cleared, and an aliquot is set aside for quality control analysis.

4. Split each sample into 300 μL aliquots and transfer to 1.5 mL Bioruptor Microtubes with caps.
5. Shear chromatin via sonication using Bioruptor Pico, set 20 cycles of 30 s ON/30 s OFF.

**Figure 2:**
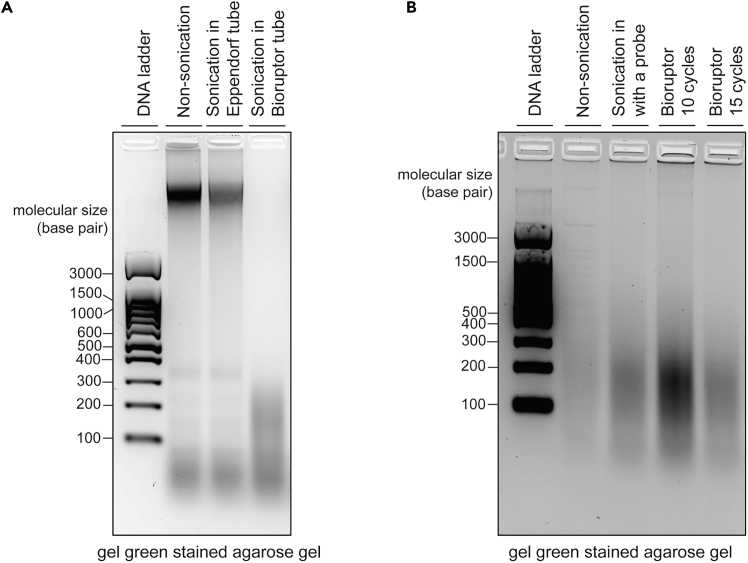
Gel of sheared DNA (A) Comparison of tube effect on sonication efficiency was tested using mouse genomic DNA in an Eppendorf or bioruptor tube. (B) Analysis of size range for sheared chromatin isolated using DNAzol and comparing the effect of a probe sonicator and bioruptor with different cycle settings.

**CRITICAL:** Bioruptor Microtubes are specifically designed for efficient delivery of sonication energy to the samples. Regular Eppendorf 1.5 mL tube will not work for this type of instrument (Figure 2A).

**CRITICAL:** When using Bioruptor tube holder, all the slots need to be filled with microtubes containing equal volumes of samples or water for similar sound wave distribution across samples.

**CRITICAL:** Sonication conditions should be determined for different sonicator model, as well as cell line or tissue type. The optimal size range of chromatin should be between 150-700 base pair.

***Alternatives:*** Here, we used diagenode Bioruptor Pico for shearing the chromatin. We also tested a probe sonicator by Cole-Parmer instruments (Model: CPX130) and had comparable result with the settings of 60% output, 1 sec pulse on/2 sec off, 15 pulses per cycle, total 4 cycles (Figure 2B).

6. Transfer samples to new 1.5 Eppendorf tubes and clear the lysate by centrifuging at 13,000 g for 10 min at 4°C. Collect and save the supernatant which contains the sheared chromatin.

***Note:*** Bioruptor tubes may break when centrifuging at high speed, therefore we use Eppendorf tubes instead for clearing samples.

7. Take an aliquot of 50 μL for assessment of chromatin shearing and store at −80°C.

***Optional:*** Save 50 μL aliquot of each sample for verifying target of interest by western blot.

**Pause point:** Chromatin can be frozen in liquid nitrogen and stored at −80°C for at least 2 months.

### Sheared chromatin quality control analysis

**Timing: Day 3, 2–3 h**

An aliquot of sheared chromatin is reverse-crosslinked and purified using PCR purification kit. The size range of chromatin is analyzed by electrophoresis. DNA concentration is measured by NanoDrop.

8. Thaw aliquoted chromatin from step 7, which contains 50 μL sheared chromatin and add 150 μL 1× ChIP elution buffer.
9. Add 8 μL 5 M NaCl to each sample, mix and then incubate at 95°C for 15 min. Afterward, cool samples to room temperature.
10. Purify and collect DNA in 50 μL of elution buffer. This protocol used the GeneJET PCR Purification Kit from Thermo Fisher (https://www.thermofisher.com/document-connect/document-connect.html?url=https://assets.thermofisher.com/TFS-Assets%2FLSG%2Fmanuals%2FMAN0012662_GeneJET_PCR_Purification_UG.pdf).
11. Use 10 μL sample and determine DNA fragment size by electrophoresis on a 1.5% agarose gel with a 100 base pair DNA marker (Troubleshooting 2).
12. To determine DNA concentration, transfer 2 μL of purified DNA to NanoDrop. The concentration of DNA should be 50–90 ng/μL.

### Chromatin immunoprecipitation—Forming antibody/chromatin complexes

**Timing: Day 3, 30 min and 16 h incubation**

An antibody (negative, positive or specific targeting protein of interest) is added to chromatin samples and incubated overnight at 4°C to allow forming antibody/chromatin complexes.

13. Into prechilled 1.5 mL Eppendorf tube, aliquot 30 μg of original sheared chromatin sample for each specific target if using multiple antibodies, plus a positive control if available and the negative control (IgG or Mock-IP).

***Note:*** To calculate amount of chromatin samples used for immunoprecipitation, we will first check the sample concentration from step 12 and then calculate then volume of samples that needed. For example, if the DNA concentration from step 12 is 100 ng/μL, then for 30 μg of sample, we should take 300 μL (30 μg × 1000/100 ng/μL = 300 μL) of original sheared chromatin.

14. Dilute samples from step 13 to final volume of 1 mL by adding proper amount of IP buffer containing 1× protease inhibitor (e.g., add 800 μL IP buffer to 200 μL chromatin sample), make sure SDS concentration is less than 0.1% after dilution.
15. Aliquot 50 μL of diluted chromatin into a new 1.5 mL tube and label as “5% Input”. Store sample at −80°C until used in step 21.
16. Add 2 μg target-specific antibody to sample, and 2 μg IgG sample for the negative control. Thus, the antibody concentration is approximately 2 μg/mL.

**CRITICAL:** When using IgG as negative control, it is important to make sure the isotope of IgG is the same as target-specific antibody.

17. Incubate IP samples overnight at 4°C with rotation.

### Chromatin immunoprecipitation—Adding beads, elute, and reverse crosslinking

**Timing: Day 4, 5 and 16 h incubation**

The antibody/chromatin complexes are pulled down by magnetic beads. Non-specific binding is largely removed by intensive washes using IP buffer. The DNA-protein complexes are eluted, and the crosslinks are reversed by heating.

18. Gently vortex ChIP-Grade Protein G magnetic beads, add 30 μL of Protein G magnetic beads to each IP reaction and incubate for 2 h, at 4°C with rotation.
19. Place the tubes in a magnetic stand, wait 1 min for solution to clear.
20. Remove supernatant carefully.
21. Wash magnetic beads with 1 mL ice-cold IP buffer.
22. Place the tubes in a magnetic stand, wait 1 min for solution to clear.
23. Remove supernatant carefully.
24. Repeat step 21 to 23 four more times.
25. Elution of chromatin and reversal of cross-links
  a. Add 150 μL of the 1× ChIP elution buffer to the 5% input sample tube from step 7, and set aside at room temperature until step 25 g.
  b. Add 150 μL 1× ChIP elution buffer to each IP sample and resuspend.
  c. Elute chromatin from the antibody/protein G magnetic beads for 30 min at 65°C with gentle vortexing (1,200 rpm).
  d. Centrifuge at 10,000 g for 10 s to remove condensed sample from cap.
  e. Pellet protein G magnetic beads by placing the tubes in a magnetic stand, wait 1 min for the solution to clear.
  f. Carefully transfer eluted chromatin supernatant to a new tube with screw cap.
    ***Optional:*** Save 10 μL aliquot of each sample verifying target of interest by immunoblot.
    **CRITICAL:** We highly recommend using tubes with screw cap for overnight reverse-crosslinking, which preserve all the samples and avoid sample loss due to evaporation.
  g. To all samples, including the “5% input” sample from step 21a, add 6 μL 5 M NaCl and 2 μL of 20 mg/mL proteinase K, and reverse cross-links by incubating at 65°C overnight.

### DNA purification and ChIP-qPCR analysis

**Timing: Day 5, 4–5 h**

Reverse-crosslinked ChIP samples are purified with a PCR purification kit. qPCR is used to evaluate the signal enrichment.

26. Centrifuge at 10,000 g for 10 s to remove condensed sample from cap.
27. Purify and collect DNA in 50 μL of elution buffer with a PCR purification kit, following manufacturer’s instructions (same as step 10).

**Pause point:** The eluted DNA products can be stored at 20°C or used right away for qPCR analysis.

28. Dilute eluted DNA 2× with nuclease free water and use 2 μL as template per qPCR reaction (Troubleshooting 3 and 4).
29. Set up PCR reaction master mix as follow. Run qPCR to evaluate the signal enrichment. Make sure to include positive and negative control primers.

**Table:** PCR reaction master mix

Reagent	Amount
DNA template	2 μL (2× diluted)
2× SYBR mix	7.5 μL
Forward primer (10 μM)	1 μL
Reverse primer (10 μM)	1 μL
ddH_2_O	3.5 μL
Total	15 μL

**Table:** PCR cycling conditions

Steps	Temperature	Time	Cycles
UDG pre-treatment	50°C	2 min	
Initial Denaturation	95°C	2 min	1
Denaturation	95°C	15 s	40 cycles
Annealing	60°C	15 s	
Extension	72°C	1 min	
Final extension	72°C	10 min	1
Hold	4°C	forever	

## Expected outcomes

Though we do not show ChIP-seq or ChIP-qPCR results in this protocol, examples of ChIP outcomes is shown in Akl et al.^1^ In this study, mouse liver nuclei was isolated, and chromatin was purified using the method described in this protocol. Enrichment of transcription factor NRF1 or NRF2 protein following anti-NRF1 or anti-NRF2 ChIP was confirmed by immunoblot (example shown in Figure 4B of Akl et al.^1^). ChIP-qPCR result shows NRF1 binds loci in genes *Psma1* and *Psmc2*, while NRF2 binds loci in genes *Ces1g* and *Gstm1* (example shown in Figure 4E of Akl et al.^1^). Moreover, ChIP-seq was used to investigate genome-wide binding sites, the results of which as well as methods used for library construction and sequencing and location of corresponding sequence data are described in Akl et al.^1^

## Limitations

Although this protocol has been successfully applied for analysis of ChIP samples from mouse liver tissue, it has not been tested for other tissues. We recommend pilot tests and optimization for each step should be considered when applying this protocol for other types of specimens. Also, the success of ChIP relies on the specificity and sensitivity of the antibody. We highly recommend using ChIP-Grade quality antibodies, if they are available.

## Troubleshooting

### Problem 1

Low DNA yield (difficult to be visible by electrophoresis from step 11 or very low reading of NanoDrop from step 12)

### Potential solution

Crosslinked samples will not completely dissolve in DNAzol reagent. Therefore, it is crucial to maintain all soluble and insoluble sample after step 3b, and do not centrifuge the sample at this step, and proceed to step 3c directly. Yield of DNA will significantly be decreased if only use the soluble portion.

Low DNA yields can also be caused by insufficient samples, especially when working with liver tissue that contains large amount of lipids (for example, livers collected from mice that have been fed high fat diet). To increase the DNA yield, more liver sample can be collected at step 2a.

### Problem 2

Inconsistent shearing of DNA (Step 5).

### Potential solution

The effect of shearing varies depending on sample type, crosslinking conditions and sonicator under use.^4^^,^^5^ When shearing the chromatin, it is important to follow the recommendations from manufacturer and optimize accordingly. For example, the Bioruptor Pico recommends not to exceed the maximum volume in the tube (related to step 4). We noticed that overfilling the tube (exceed the maximum limit of volume) reduce the efficiency of sonication. It is also important to ensure sample holder is spinning when sonication cycle starts, otherwise the sonication will not be efficient and consistent.

We recommend optimizing sonication conditions when using a probe sonicator (such as CPX130 from Cole-Parmer instruments), for example, by testing a time course with a starting output of 50%. For duration of sonication, a series of short pulses is more efficient than a single long pulse,^6^ which also produce less heat during sonication.

### Problem 3

High background noise of ChIP-qPCR (Step 25).

### Potential solution

High specificity and sensitivity of antibody is key to success of ChIP experiments. Using a ChIP-Grade quality antibody will reduce background noise and improve signal to noise ratio (step 16). Increase the time and repetition of washing steps. Alternatively, beads can be washed with more stringent buffer (e.g., IP buffer contains 400 μM sodium chloride) and prolong washing time to further remove non-specific binding (step 20).

### Problem 4

Low ChIP signal (Step 25).

### Potential solution

Low ChIP signal, or enrichment of positive control region is not different than IgG negative control, can result for multiple reasons. Potential solutions are as follows.

• Conducting a pilot experiment including a positive control ChIP using ChIP-grade antibody against a protein that strongly binds to chromatin, such as anti-histone 3.
• Take aliquots at step 3, 7 and 21 and determine the enrichment of target of interest at each step by western blotting. If less protein is enriched after chromatin immunoprecipitation, increase the amount of antibody used or try different antibody.
• Less stringent washes may be used, but care need to be taken that this will also increase the background noise.
• It is possible that target of interest may interact with DNA transiently and cannot be efficiently detected by ChIP,^7^ or the formaldehyde crosslinking method may not be sufficient for target of interest. For hyper-dynamic transcription factors, combination of protein-protein fixation followed by protein-DNA fixation can be used.^8^^,^^9^

## Resource availability

### Lead contact

Further information and requests for resources and reagents should be directed to and will be fulfilled by the lead contact, Scott B. Widenmaier (scott.widenmaier@usask.ca).

### Technical contact

Further information regarding specific details of the steps for this protocol should be directed to and will be fulfilled by the technical contact, Lei Li (lei.li@usask.ca).

### Materials availability

This study did not generate any unique reagents or datasets.

### Data and code availability

The ChIP-Seq data generated using this protocol has been published^1^ and is available at the National Center for Biotechnology Information repository (NCBI; BioProject ID: PRJNA806849).

## Acknowledgments

We thank Austin Hammond as well as Mohan Babu and colleagues for experimental assistance with ChIP-seq and associated analysis. This work was supported by a Saskatchewan Health Research Foundation Establishment grant and Canadian Institutes of Health Research Project grant. The graphical abstract was created with Biorender.com.

## Author contributions

L.L. optimized the protocol and wrote the original manuscript. M.G.A. contributed to optimizing and collecting mouse liver sample. S.B.W. conceived the approach for the protocol, contributed to optimizing and collecting mouse liver sample, edited the manuscript, and supervised the project.

## Declaration of interests

The authors declare no competing interests.
